# Supplementary material for: A Monte-Carlo planning strategy for medical follow-up optimization: Illustration on multiple myeloma data
Source: PLoS One. 2024 Dec 19;19(12):e0315661. doi: 10.1371/journal.pone.0315661 (PMC11658637; doi:10.1371/journal.pone.0315661)
Supplement: S1 File — (PDF) [file pone.0315661.s001.pdf]

Supporting information

State of the art

Artificial Intelligence in medicine The use of artificial intelligence methods in medicine has recently exploded, as was shown for example in [48]. Yet the vast majority of these works focus on diagnosis and prognosis, rather than treatment and follow-up.

There are a few studies related to treatment of diseases, though. [49] provides a review of approaches to cancer treatment focused on medical decision support. In the context of drug design for cancer treatment, (deep) Reinforcement Learning (RL) approaches have been proposed recently [50, 51], but with the disadvantage of being black-box approaches, preventing the practitioner from access to an explainable model of disease evolution and treatment. Uncertainty quantification in models of assisted decision making have been proposed to alleviate this problem [52]. In the same line of work, [53] advocate learning of mechanistic models of cancer evolution/treatment in order to help decision making. However, the latter approach requires a large amount of data prior to applying any treatment action, in order to learn a model that may prove only partially valid as decisions influence the disease dynamics. The authors advocate that the approach may be rendered more efficient if learning phases are interleaved with actual decision phases.

An alternative view of controlled PDMPs Controlled PDMPs can be modeled as continuous space POMDPs, in the way we proposed in this paper. However, they can also be seen as a particular subclass of continuous-time (Partially Observed) Semi-Markov Decision Processes [54]. Fully-observed continuous-time Semi-Markov Decision Processes extend Markov Decision Processes by including random continuous durations of state transitions and by considering that decisions can only be made at transition times. Several reinforcement learning solution approaches have been proposed, both in the fully-observed [55, 56] and partially-observed [57] cases to solve these problems. An alternative approach to ours could be to cast the PDMP model of cancer treatment into the continuous-time SMDP model and look for specializations of the existing simulation based solution algorithms.

Supplementary simulation results on POMCP parameters

Here we provide raw results for a series of parameter we tried to tune to optimize the POMCP algorithm, but that did not seem to bring additional improvement in our framework. Table 4 shows the impact of the trade off parameter α' in a few scenarios for the particle filter.

To allow adaptive selection of the trade off parameter c we tried three dynamic procedures to exploit or explore more depending on our trust in the current patient state. To do so, we define the state entropy as $E_t = \sum_{m=0}^{2} p_m \log(p_m)$, $p_j = \sum_{s=(m,\zeta,u)\in B} \mathbf{1}\{m = j\}$ and $E_{\max} = \log(1/3)$ and consider the following:

- entropy: $\alpha_t = E_t/E_{\max}$
- rev-entropy: $\alpha_t = 1 - E_t/E_{\max}$
- rev-entropy-2: $\alpha_t = 1 - E_t/2E_{\max}$.

However, none of those procedures improved the results, as illustrated in selected examples in Table 5.

Table 4. Raw results for the particle filter varying parameters α', n_{search} and K. For each parameter set, $n = 500$ trajectories were simulated. The Value column is the average cost of the trajectories over the n trajectories, and $\hat{\sigma}$ its empirical variance. We also recorded the runtime of optimizing each trajectory (duration column).

Filter	$\pi_{rollout}$	n_{search}	K	α'	Value	$1.96\hat{\sigma}/\sqrt{n}$	duration	duration s.d
particle	π_{mode}	100	100	0.2	161.93	14.79	1730	982
particle	π_{mode}	100	100	0.5	156.01	13.01	1629	913
particle	π_{mode}	100	100	0.8	165.63	13.51	1714	1053
particle	π_{mode}	100	100	0.99	147.24	6.17	1641	971
particle	π_{mode}	100	500	0.2	141.10	9.83	2727	683
particle	π_{mode}	100	500	0.5	141.56	4.72	2771	699
particle	π_{mode}	100	500	0.8	134.98	4.44	2646	685
particle	π_{mode}	100	500	0.99	133.57	3.56	2640	611
particle	π_{mode}	500	100	0.2	146.30	8.27	4617	660
particle	π_{mode}	500	100	0.5	146.82	11.91	4047	670
particle	π_{mode}	500	100	0.8	145.87	13.01	3708	610
particle	π_{mode}	500	100	0.99	140.91	6.35	3454	614
particle	π_{mode}	500	500	0.2	135.99	4.00	5182	598
particle	π_{mode}	500	500	0.5	132.88	4.25	5068	643
particle	π_{mode}	500	500	0.8	136.14	8.08	5271	698
particle	π_{mode}	500	500	0.99	129.42	5.06	4946	724

Table 5. Simulation results with adaptive choice of the exploration/exploitation parameter α'. For each parameter set, $n = 500$ trajectories were simulated. The Value column is the average cost of the trajectories over the n trajectories, and $\hat{\sigma}$ its empirical variance. We also recorded the runtime of optimizing each trajectory (duration column).

Filter	n_{search}	α'	Value	$1.96\hat{\sigma}/\sqrt{n}$	duration
conditional	100	entropy	138.63	5.06	776
conditional	100	rev-entropy	131.94	3.70	786
conditional	100	rev-entropy-2	133.75	3.70	770
particles	100	entropy	142.17	9.88	2421
particles	100	rev-entropy	143.27	10.34	2473
particles	100	rev-entropy-2	135.28	3.82	2438
conditional	1000	entropy	131.78	4.70	8313
conditional	1000	rev-entropy	131.41	3.45	8432
conditional	1000	rev-entropy-2	132.73	3.56	8332
particles	1000	entropy	133.39	3.74	9994
particles	1000	rev-entropy	135.64	3.74	10047
particles	1000	rev-entropy-2	131.89	4.24	10028

References

48. Kumar Y, Koul A, Singla R, Ijaz MF. Artificial intelligence in disease diagnosis: a systematic literature review, synthesizing framework and future research agenda. Journal of Ambient Intelligence and Humanized Computing. 2022; p. 1–28.
49. Bhinder B, Gilvary C, Madhukar NS, Elemento O. Artificial intelligence in cancer research and precision medicine. Cancer discovery. 2021;11(4):900–915.
50. Olivecrona M, Blaschke T, Engkvist O, Chen H. Molecular de-novo design through deep reinforcement learning. Journal of cheminformatics. 2017;9(1):1–14.
51. Popova M, Isayev O, Tropsha A. Deep reinforcement learning for de novo drug design. Science advances. 2018;4(7):eaap7885.
52. Begoli E, Bhattacharya T, Kusnezov D. The need for uncertainty quantification in machine-assisted medical decision making. Nature Machine Intelligence. 2019;1(1):20–23.
53. Benzekry S. Artificial intelligence and mechanistic modeling for clinical decision making in oncology. Clinical Pharmacology & Therapeutics. 2020;108(3):471–486.
54. Howard R. Semi-markovian decision processes. Bulletin de l'Institut International de Statistique. 1964;40(1).
55. Bradtke SJ, Duff MO. Reinforcement learning methods for continuous-time markov decision problems. In: Advances in neural information processing systems; 1995. p. 393–400.
56. Doya K. Reinforcement learning in continuous time and space. Neural computation. 2000;12(1):219–245.
57. Du J, Futoma J, Doshi-Velez F. Model-based Reinforcement Learning for Semi-Markov Decision Processes with Neural ODEs; 2020.
